# Stride Pattern of the Lower Extremities Among Stride Types in Baseball Pitching

**DOI:** 10.3389/fspor.2021.670395

**Published:** 2021-08-02

**Authors:** Shu-Wei Chen, Wen-Tzu Tang, Jung-Tang Kung, Tsung-Ying Hung, Yu-Lin Chen, Wei-Hsuan Lin, Daniel J. Burgee

**Affiliations:** ^1^Graduate Institute of Athletics and Coaching Science, National Taiwan Sport University, Taoyuan City, Taiwan; ^2^Department of Sports Training Science-Balls, National Taiwan Sport University, Taoyuan City, Taiwan; ^3^Department of Leisure Sports and Health Management, St. John's University, New Taipei City, Taiwan

**Keywords:** kinetic chain, movement pattern, overhead throwing, pivot leg, stride leg

## Abstract

The present study investigated the differences in the stride pattern of the lower extremities among different stride types in baseball pitchers with the aim of evaluating stride movement and skills to improve training effectiveness. Thirty elite male college baseball pitchers volunteered to pitch on an indoor-mound-like force plate, where motion data of their fastest strike trials were collected using an eight-camera motion analysis system at a 200–250 Hz sampling rate. Pelvis center trajectories of each participant were calculated and further categorized into three groups: tall-and-fall (TF), dip-and-drive (DD), and mixed (MX) pitchers. Motion analysis revealed that DD pitchers initiated pivot–knee extension and pivot–hip adduction earlier than TF pitchers and accelerated their bodies sooner than TF pitchers. In addition, TF pitchers accelerated their bodies forward by pivoting their legs until the middle of the arm-cocking and acceleration phases. The movement patterns of MX pitchers were similar to those of DD pitchers in terms of pivot leg, although this occurred a little later in the stride. Our findings are useful in developing training strategies for coaches, players, and trainers to better meet the demands of different pitching styles.

## Introduction

Many coaches believe that pitchers are taught pitching in different ways due to differences in body size, physical characteristics, and baseball culture (Osinski, 1998; Thurston, 1998). This is especially true in Asian countries (including Korea and Japan) and countries in the Americas, which has led to different pitching movements being observed in these countries (Escamilla et al., 2002; Oi et al., 2019). Several studies have focused on the different pitching mechanics among various countries (Escamilla et al., 2001, 2002; Dowling et al., 2017; Oi et al., 2019), especially the pitching performances and biomechanics of American and Japanese pitchers. The previous research stated that Japanese and American coaches instruct different pitching movements for the lower body (Oi et al., 2019). This research mentioned that American pitchers are taught to stride forward during the stride phase of pitching and then to extend their lead knee after landing to use the lead leg as a stable base to rotate the trunk. Also, they showed that the extending lead knee helps brace and stabilize the lead leg; this may enhance the ability of the trunk to more effectively rotate forward over the braced lead leg. Furthermore, Japanese pitchers are taught to lower their center of mass and stride out, moving the trunk forward toward the lead foot, and keeping their front knee flexed during ball release. However, the hip and ankle joints, which are the adjacent joints of the knee joint, may also be different, but have not been investigated. Besides, it is unknown how kinematic differences between the stride types are more related to pitcher anthropometry or pitching pattern.

**Figure 1:**
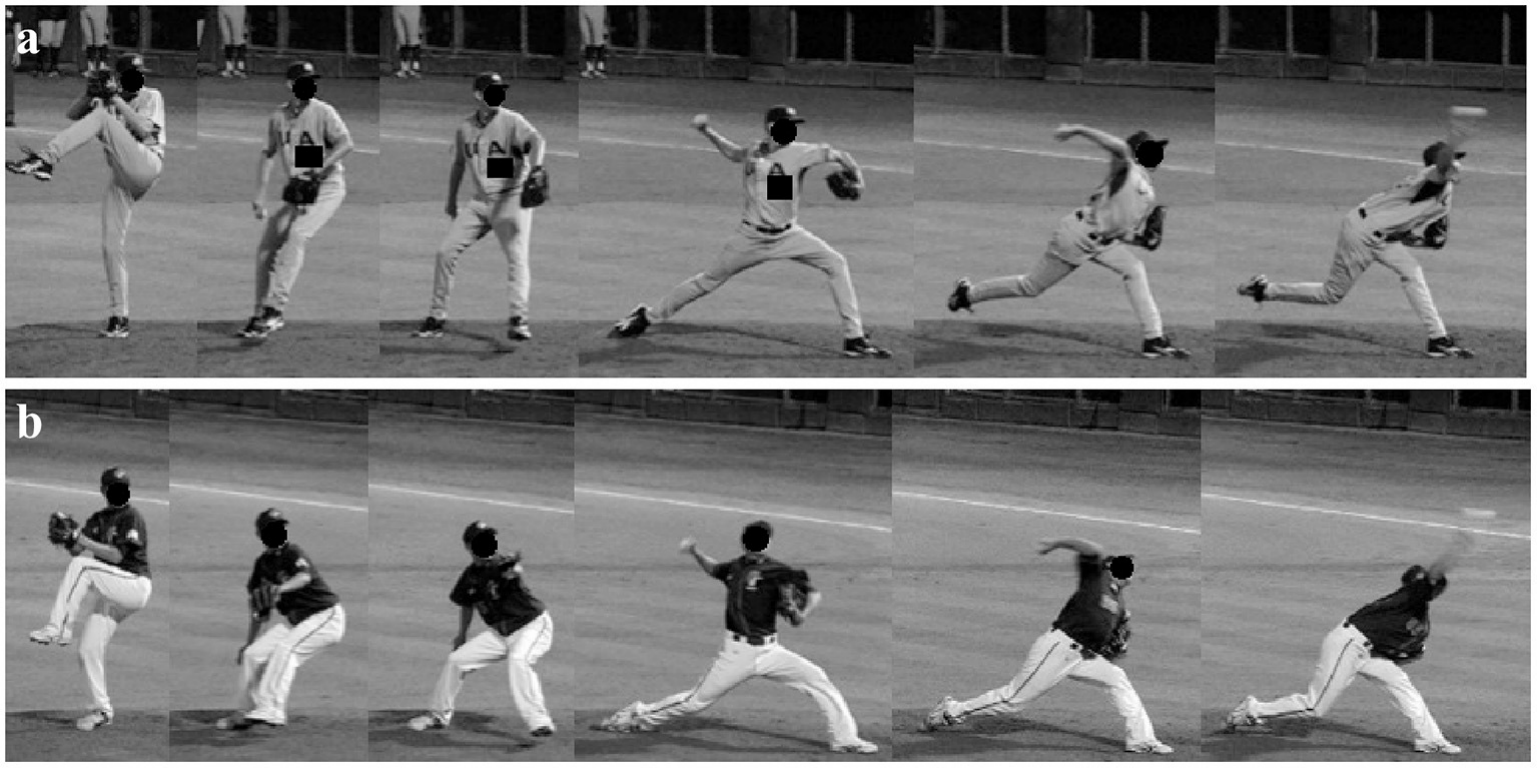
Time-lapse photographs of the pitching motion of a typical tall-and-fall-type pitcher **(a)** and a typical dip-and-drive-type pitcher **(b)**.

Some players and coaches also suggested that there were two different lower extremity stride types in baseball pitching: tall-and-fall (TF) and dip-and-drive (DD) (Ryan and House, 1991). Pitchers categorized as TF pitchers keep their body as tall as possible and release the ball when it is at the highest position during their movement. DD pitchers, however, frequently bend down their knee to drive or push off the rubber, similar to how Japanese pitchers gain momentum (Figure 1) (Oi et al., 2019). DD pitching thus relies on the press force of the pivot leg, whereas TF pitching depends on the muscle strength of the trunk and throwing arm (Ryan and House, 1991; Chen et al., 2011). This kind of movement (dipping down a lot to drive) also lowers the pelvis first before the drive, an approach similar to that used by Japanese pitchers (Oi et al., 2019). When studying the biomechanical effects of the two stride types on the pivot leg of elite Taiwanese baseball pitchers, the previous research found that the timing and amount of knee flexion angle and ground reaction force in the pivot leg during the stride phase were the key differences between the TF and DD groups (Chen et al., 2011). Combining these studies, the movement trajectory of the pelvis on the sagittal plane (forward-downward dimension) should be the classification basis of different stride types, and the movement of pivot leg to drive forward and the movement of stride leg after landing would be different, however, were not investigated yet.

Taiwanese baseball players are fully aware of the Japanese and American roots of the game. Baseball was introduced to Taiwan during the Japanese period of colonization (1895–1945); therefore, Japanese training culture became the tradition of coaches in Taiwan (Morris, 2017). But in these 30 years, American baseball players and coaches who played on Taiwanese professional baseball teams also introduced American training culture to Taiwan (Jordan et al., 2004). Therefore, Taiwanese baseball training culture is influenced by both Japan and America, where the specific practices and characteristics of Japanese and American pitchers could be found in Taiwanese pitchers. As players often exhibit different body types, e.g., as seen in Asian and American cultures, it is useful to compare the TF and DD striding types among players with similar anthropometry body types from the same country or region to investigate the kinematic differences in the two striding types in relation to body size and height control.

The lower extremities play a very important role during pitching in baseball. The movement of the lower extremities accounts for almost half of the throwing velocity through foot stride and trunk rotation (Toyoshima et al., 1974) with a high muscle activity level (Yamanouchi, 1998; Campbell et al., 2010). Previous study also suggested that lower-extremity kinematics might affect performance and risk of injuries during pitching (Kung et al., 2017). Matsuo et al. (2001) also suggested that stabling leg during stride knee extension helps to transfer energy through the trunk to the throwing arm, which might be critical for maximizing pitch velocity. Together, movements of the lower extremities during pitching are highly associated with pitching performance and injury risk. In order to gain an insight into stride patterns and skills that could be used to improve training, the purpose of the study is to examine the stride types and pelvis movement patterns while further investigating the differences in stride movement patterns of lower limbs among baseball pitchers with disparate stride types. Besides, our study is the first of its kind to compare different stride techniques with similar anthropometry through hip, knee, and ankle angular kinematics. We hypothesized that different stride patterns and skills in terms of hip, knee, and ankle angular kinematic and timing variables would be observed among the pitchers with disparate stride types because of the unique movements of their lower extremities. Specifically, the DD pitchers would generate more drive or push movements with more extension by pivot leg during the stride phase and would generate less bracing movements with more flexion by stride leg during the arm-cocking and acceleration phase.

## Methods

### Participants

Thirty elite male Taiwanese collegiate and professional baseball pitchers (mean age of 21.17 ± 2.83 years, height of 1.80 ± 0.07 m, weight of 81.20 ± 10.18 kg, and maximum ball velocity of 39.22 ± 2.06 m/s) volunteered to participate in this study. The study and all of its protocols were approved by the Institutional Review Board of National Taiwan Sports University (NTSUIRB-S980302-01). After an explanation that was given for the purpose of this study and associated risks, all participants provided a written informed consent to participate.

### Experimental Setup and Procedures

A radar gun (Stalker Sport, Stalker Radar/Applied Concepts Inc., Richardson, TX, United States) was used to measure ball velocity, and kinematic variables were measured using the Eagle System with eight Eagle cameras (Motion Analysis Corporation, Santa Rosa, CA, United States), at a sampling rate of 200 or 250 Hz. Two force plates (BP600900 and BP400600, Advanced Management Technology Inc., Watertown, MA, United States) with a 1,000 or 1,250 Hz sampling rate were used to identify the instant that the stride foot made contact with the ground. To simulate a real pitching situation, an indoor-mound-like force plate setting was used. The force plate that recorded the ground reaction force of the pivot leg (AMTI BP400600, 40 cm × 60 cm) was mounted 10 cm above and 65 cm behind the force plate that recorded the ground reaction force of the stride leg (AMTI BP600900, 60 cm × 90 cm), in line with the baseball rulebook (Chen, 2007). The force plates were needed to maintain horizontal, unlike the slope of a regulation pitching mound at the landing place of stride foot. Due to the use of force plates, the participants could not wear spikes as usual; only tight shorts, socks, and sport shoes could be worn by participants. A total of 42 reflective markers (12 mm in diameter for the head, shoulder, and pelvis; 8 mm in diameter for other locations) were placed on body landmarks of the participants to define the joint centers, body segments, and joint coordinate systems. The marker set and segments configuration was using a combination of the modified Helen Hayes marker set (Liu et al., 2021) and an upper-body marker set (Aguinaldo et al., 2007), referenced from the manual of KinTools RT for Cortex 1.1.4 software. The following markers were used to define the pelvis and lower extremities: both sides (left and right) of anterior superior iliac spine (ASIS), posterior superior iliac spine (PSIS), trochanter major, epicondyle of femur (lateral and medial), lateral epicondyle of fibula, medial epicondyle of tibia, third metatarsi, and calcaneus. Using the ASIS breadth as the reference, the hip joint center was determined by moving 32% laterally, 22% posteriorly, and 34% inferiorly. The knee joint center was determined by the midpoint of the lateral epicondyle of femur and the medial epicondyle of femur, and the ankle joint center was determined by the midpoint of the lateral epicondyle of fibula and the medial epicondyle of tibia. The foot segment was defined by the ankle joint center and third metatarsi marker. Additionally, the rest of the markers were placed to define head, trunk, upper arm, forearm, and hand segments. The root mean square error in the calculation of three-dimensional marker location was <0.5 mm. Because of limitations in the space of the experiment, a pitching net indicating virtual strike zone (marked by tape) was set 7.5 m in front of the pitching mound, and the size of strike zone was also set with the baseball rulebook, assuming that the height of batter was 180 cm. After personal parameters (age, height, weight, medical history, and health status) were recorded, participants were asked to warm up using their personal routine. Participants were then asked to throw three strike pitches at the maximum effort, where data on the trial of the fastest pitch were used for analysis.

### Data Collection and Analysis

The three-dimensional coordinates and the GRF were synchronized using Cortex v.1.1.4 software. Marker position data were filtered using a fourth-order Butterworth low-pass filter with a cutoff frequency of 13.4 Hz (Escamilla et al., 1998) using Matlab v2010a (MathWorks, Inc., Natick, MA, United States). Based on prior definitions of the pitching motion, this activity was separated into six phases: wind-up, stride, arm-cocking, arm acceleration, arm deceleration, and follow-through (Fleisig et al., 2006). The pitching movement from where the knee of the stride leg reaches peak height to the point when the stride foot contacts the ground was defined as stride phase, and the pitching movement from the stride foot contacting the ground to the instant of ball release was defined as arm cocking and acceleration phase. The moment of stride foot contact was defined by the resultant force of the stride leg that was >50 N. All parameters and variables that were evaluated are detailed in Table 1. The stride length was defined by the distance between the two joint centers of the ankle when the stride foot contacted the ground (Fleisig et al., 2006; Milewski et al., 2012), and it was expressed as a percentage of participant height. The projection angles of lower extremity—including the hip internal/external, abduction/adduction, and extension/flexion angles; the knee extension angle; and the ankle dorsi-flexion angle—were calculated as described by previous study (Wu et al., 2002). All temporal variables are presented as the relative time percentage normalized by either the duration of the stride phase or the duration of the arm-cocking and acceleration phase.

**Table 1:** List of all parameters and variables evaluated in the current study.

**Categories**	**Variables**
Ball-velocity parameters	Maximum in the current study (m/s)
Stride parameters	Stride/length ratio^*^ (body height %)
Temporal parameters	Duration of the stride phase and arm-cocking and acceleration phase(s)
	Timing of peak angle/angular velocity in the stride phase and arm-cocking and acceleration phase (time %)
Angular parameters^**^	Angle/angular velocity at SFC and ball release (° and °/s)
	Peak value of angle/angular velocity in the stride phase and arm cocking and acceleration phase (° and °/s)

* *The stride length was defined by the distance between the two joint centers of the ankle when the stride foot contacted the ground, and it was expressed as a percentage of participant height*.

** *Included hip internal/external rotation, hip abduction/adduction, hip extension/flexion, knee extension/flexion, and ankle plantar/dorsi-flexion of lower extremities (pivot leg and stride leg)*.

*SFC, stride–foot contact*.

### Grouping of Participants

**Figure 2:**
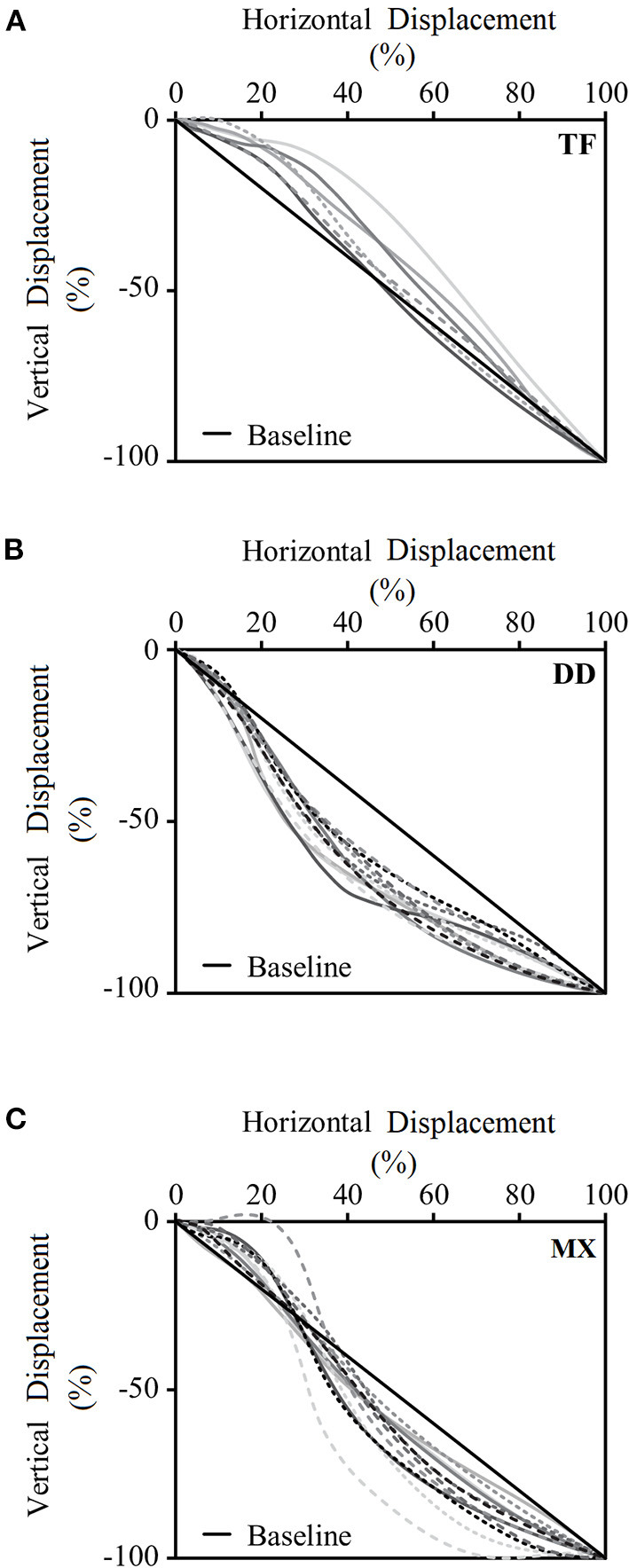
Pelvis center trajectories in the stride phase. The horizontal axis of the figure represents the standardized pelvis center movement in the X-axis (forward-pitching direction) during the stride phase. The vertical axis of the figure represents the standardized pelvis center movement in the Z-axis (vertical direction) during the stride phase. Graphs illustrate the pelvis center trajectories of **(A)** tall-and-fall pitchers, **(B)** dip-and-drive pitchers, and **(C)** mixed pitchers. TF, tall-and-fall pitchers; DD, dip-and-drive pitchers; MX, mixed pitchers.

The pelvis center was calculated as the average position of the two (right and left) ASIS markers and the two (right and left) PSIS markers. It was calculated in order to assign the participants into different groups based on the definitions of stride types (Ryan and House, 1991). The pelvis center displacement was standardized by the pelvis maximum value of displacement in each component of X and Y, respectively. Two investigators determined groups based on the pelvis center trajectories in the stride phase. Cohen's Kappa was applied to detect the existence of agreements by chance. Both inter- and intra-observer reliability exhibited Cohen's Kappa for each observational category coefficient as 1.00. If the trajectory of the pelvis center moved forward first and then downward (i.e., most of curves showed above the slope), then the participant was identified as a TF pitcher; if the trajectory of the pelvis center moved downward first and then forward (i.e., most of curves showed below the slope), then the participant was identified as a near DD pitcher; if the trajectory of the pelvis center moved forward first and then quickly downward (i.e., the curves showed above over portion 20% horizontal displacement and then below the slope), then the participant was considered as a mixed (MX) pitcher (Figure 2).

### Statistical Analysis

Statistical analysis of each variable across the two pitching phases was performed individually for the three stride types using SPSS v17.0 (IBM Corporation, Armonk, NY, United States). Data were presented as means ± standard deviation, and statistical significance was accepted at *p* < 0.05. All data were checked for normality with the Shapiro–Wilk test. To compare differences among stride types, normally distributed variables were first assessed from the one-way ANOVA to identify differences among the three stride types, then based on equality of variances with a Scheffe *post-hoc* test or non-equality with a Games–Howell *post-hoc* test. Non-normally distributed variables were evaluated with the independent Kruskal–Wallis H-test to identify the differences among the three stride types, then with Dunn's *post-hoc* test.

## Results

All parameters and variables that were evaluated in the current study are shown in Table 1. No significant differences were observed among the three stride groups in terms of ball velocity, stride, or temporal variables (Table 2). The statistical summary of lower-extremity parameters at selected events (Table 3) showed that only the hip adduction angular velocity and ankle plantar–flexion angular velocity of the pivot leg were significantly different among the three groups at the moment of stride foot contacted the ground. The statistical summary of lower-extremity parameters during the stride phases and the arm-cocking and acceleration phase (Table 4) showed that during the stride phase, there were significant differences in hip external rotation (peak angular velocity), hip adduction (peak angular velocity), hip extension (peak time of angle, timing of peak angular velocity), knee (timing of peak flexion angular velocity and peak extension angle), and ankle (peak angular velocity of plantar–flexion, timing of peak dorsi-flexion angular velocity). And during the arm-cocking and acceleration phase, a significant difference was also found in hip adduction (timing of peak angular velocity). A comparison of the lower-extremity parameters that exhibit significant differences between the three stride types is shown in Table 5. The DD pitchers were significantly faster in pivot hip (external rotation, adduction) and pivot knee (extension) angular velocity than TF pitchers during the stride phase. The DD pitchers also extended their pivot hip, knee, and ankle earlier than TF pitchers during the stride phase. Otherwise, the MX pitchers were significantly faster in pivot hip (adduction) and pivot ankle (plantar–flexion) angular velocity than TF pitchers. The MX pitchers also extended their pivot knee earlier than TF pitchers, but later than DD pitchers during the stride phase. Finally, the TF pitchers were significantly later to keep adducting their pivot hip than the MX pitchers during the arm-cocking and acceleration phase. The remaining variables related to the pivot and stride leg angles were not significantly different between the three stride types.

**Table 2:** Comparison of ball velocity, stride, and temporal parameters among the three stride types.

**Variables**	**TF group (*n* = 6)**	**DD group (*n* = 12)**	**MX group (*n* = 12)**	***p***	**F value/effect size**	**H value/effect size**
Ball-velocity parameters						
Max in the current study (m/s)	34.81 ± 1.84	34.06 ± 2.48	33.13 ± 2.55	0.355	0.068/0.009	
Stride parameters						
Stride/length ratio (body height %)	68.99 ± 4.57	67.86 ± 4.62	64.81 ± 3.84	0.107	0.336/0.115	
Basic event temporal parameters						
Duration of stride phase (s)	0.78 ± 0.22	0.78 ± 0.11	0.72 ± 0.14	0.485	0.239/0.317	
Duration of arm cocking and acceleration phase(s)	0.19 ± 0.03	0.16 ± 0.03	0.18 ± 0.02	0.218	0.425/0.145	

*TF, tall-and-fall; DD, dip-and-drive; MX, mixed*.

**Table 3:** Statistical summary of joint angle and joint angular velocity at selected events.

**Significantly different**	**Stride–foot contact**		**Ball release**	
	**Pivot leg**	**Stride leg**	**Pivot leg**	**Stride leg**
Hip angle				
Internal/external rotation	–	–	–	–
Abduction/adduction	–	–	–	–
Extension/flexion	–	–	–	–
Knee angle				
Extension/flexion	–	–	–	–
Ankle angle				
Plantar-/dorsi-flexion	–	–	–	–
Hip angular velocity				
Internal/external rotation	–	–	–	–
Abduction/adduction	+ DD>TF	–	–	–
Extension/flexion	–	–	–	–
Knee angular velocity				
Extension/flexion	–	–	–	–
Ankle angular velocity				
Plantar-/dorsi-flexion	+ MX>TF	–	–	–

*+: Significantly different among groups; –: not significantly different among groups*.

**Table 4:** Statistical summary of joint angle and joint angular velocity during the stride and arm-cocking and acceleration phases.

**Significantly different**	**Peak value**				**Peak time**			
	**Stride phase**		**Arm-cocking and acceleration phase**		**Stride phase**		**Arm-cocking and acceleration phase**	
	**Pivot leg**	**Stride leg**	**Pivot leg**	**Stride leg**	**Pivot leg**	**Stride leg**	**Pivot leg**	**Stride leg**
Hip angle								
Internal/external rotation	–		–	–	–		–	–
Abduction/adduction	–		–	–	–		–	–
Extension/flexion	–		–	–	+ TF>DD		–	–
Knee angle								
Extension/flexion	–		–	–	+ TF>DD TF>MX MX>DD		–	–
Ankle angle								
Plantar/dorsi-flexion	–		–	–	–		–	–
Hip angular velocity								
Internal/external rotation	+ DD>TF		–	–	–		–	–
Abduction/adduction	+ DD>TF MX>TF		–	–	–		+ TF>MX	–
Extension/flexion	–		–	–	+ TF>DD		–	–
Knee angular velocity								
Extension/flexion	+ DD>TF MX>TF		–	–	+ MX>DD		–	–
Ankle angular velocity								
Plantar/dorsi-flexion	+ MX>TF		–	–	+ TF>DD		–	–

*+: Significantly different among groups; –: Not significantly different among groups*.

**Table 5:** Comparison of the lower-extremity angle among the three stride types.

**Variables**	**TF group (*n* = 6)**	**DD group (*n* = 12)**	**MX group (*n* = 12)**	***p*/Comparison**	**F value/effect size**	**H value/effect size**
Pivot hip external rotation						
Peak angular velocity in stride phase (°/s)	160.21 ± 45.09	307.21 ± 139.87	233.38 ± 144.66	0.011^*^DD>TF		6.052/0.209
Pivot hip adduction						
Angular velocity at SFC (°/s)	66.20 ± 41.81	166.95 ± 107.07	154.83 ± 116.46	0.014^*^	2.087/0.134	
				DD>TF		
Peak angular velocity in stride phase (°/s)	78.84 ± 34.68	177.15 ± 88.15	168.68 ± 105.02	0.004^*^		5.465/0.188
				DD>TF MX>TF		
Timing of peak angular velocity in arm-cocking and acceleration phase (%)	155.46 ± 19.77	132.12 ± 15.98	131.45 ± 19.67	0.029^*^	4.030/0.230	
				TF>MX		
Pivot hip extension/flexion						
Timing of peak flexion angle in stride phase (%)	79.05 ± 3.69	68.20 ± 10.12	73.30 ± 4.92	0.021^*^		8.618/0.297
				TF>DD		
Timing of peak extension angular velocity in stride phase (%)	99.64 ± 0.61	96.79 ± 3.62	97.88 ± 2.28	0.011^*^		4.025/0.139
				TF>DD		
Pivot knee extension/flexion						
Timing of peak flexion angular velocity in stride phase (%)	42.38 ± 26.53	23.24 ± 16.80	42.23 ± 14.88	0.029^*^	3.743/0.217	
				MX>DD		
Timing of peak flexion angle in stride phase (%)	89.65 ± 7.58	69.43 ± 7.92	77.28 ± 3.84	0.000^*^	19.354/0.589	
				TF>DD TF>MX MX>DD		
Peak extension angular velocity in stride phase (°/s)	121.97 ± 40.98	211.16 ± 103.59	221.00 ± 59.16	0.002^*^	3.312/0.197	
				DD>TF MX>TF		
Pivot ankle plantar flexion/dorsi-flexion						
Angular velocity of plantar flexion at SFC (°/s)	225.39 ± 152.31	345.17 ± 107.45	388.93 ± 127.04	0.043^*^	3.168/0.190	
				MX>TF		
Peak angular velocity of plantar flexion in stride phase (°/s)	225.39 ± 152.31	368.46 ± 112.07	393.73 ± 124.90	0.033^*^	3.692/0.215	
				MX>TF		
Timing of peak dorsi-flexion angular velocity in stride phase (%)	54.87 ± 16.58	24.17 ± 20.39	38.29 ± 21.37	0.017^*^		7.078/0.244
				TF>DD		

* *P < 0.05*.

*TF, tall-and-fall; DD, dip-and-drive; MX, mixed; SFC, stride–foot contact*.

## Discussion

### Comparison With Other Studies in the Laboratory for Kinematics

The pitching performances for ball velocity were similar for all three stride groups in the current study. Previous studies have reported a ball velocity of 35 m/s among collegiate pitchers (Escamilla et al., 1998; Fleisig et al., 1999, 2006; Kageyama et al., 2014), which is similar to the velocities observed in the current study. The stride/length ratio reported in previous studies ranges from 70 to 85% of the total body height (Escamilla et al., 1998; Fleisig et al., 1999, 2006; Kageyama et al., 2014). While this range indicates slightly longer stride lengths than those observed in the current study, we recorded a stride/length ratio similar to the 69% reported for adolescent pitchers (Milewski et al., 2012) and the 70% reported for collegiate pitchers (Fleisig et al., 2006). These data suggest that participants of these two studies wore their own running shoes to pitch on indoor-mound-like force plates without pitching rubber and did not replicate their usual pitching stance on the mound.

### The Different Stride Pattern in the Lower Extremities

We found that the earlier pushing movement (hip and knee extension) of the pivot leg of DD pitchers during the stride phase was compared with that of TF pitchers (Table 5). These results demonstrate that DD pitchers initiated pivot hip and knee extension (i.e., right after hip and knee maximum flexion angle) earlier to drive body forward in the stride phase both around 70% of the stride phase, whereas TF pitchers started pivot hip and knee extension during the driving forward movement when the stride foot was close to contacting the ground (hip around 80% and knee around 90% of the stride phase). The movement patterns of MX pitchers were similar to those of DD pitchers in terms of the pivot leg, except that the forward movement was initiated a little later. These findings are similar to those presented in the textbook (Ryan and House, 1991), confirming the existence of different TF and DD stride types. Furthermore, our results support those of previous studies (Chen et al., 2010, 2011) that suggested the key difference between TF and DD stride types is the timing of the pivot leg as it begins to drive the body forward.

### The Mechanism of the Kinematic Differences Among Three Stride Types in Pivot Leg

We hypothesized that the DD pitchers would generate more drive or push movements with more extension by pivot leg during the stride phase, and we found that the DD pitchers drove their pivot leg faster (faster angular velocities of hip external rotation, knee extension, and ankle plantar–flexion) than TF pitchers. Japanese pitchers can generate greater momentum by hip external rotation, hip abduction, hip extension, and knee extension in the pivot leg for accelerating the body forward (Kageyama et al., 2014). Therefore, the stride pattern of DD pitchers in pivot leg was similar to that of the Japanese pitchers (Dowling et al., 2017; Oi et al., 2019). Otherwise, the forward acceleration of bodies of TF pitchers could be maintained through pivot–hip extension and pivot–knee extension after the stride leg has contacted the ground, where the stable stride leg is used to brace the body. This kind of stride pattern of TF pitchers in pivot leg was similar to that of the American pitchers (Dowling et al., 2017; Oi et al., 2019). It was worth noting that the DD pitchers adduct their pivot–hip faster near SFC. To rotate their pelvis, the pitchers have to adduct their pivot–hip to reduce the pelvis moment of inertia. Japanese pitchers rotate their pelvis earlier than American pitchers (Oi et al., 2019), and it may suggest that DD pitchers rotate their pelvis earlier, too. The timing of pelvis rotation during pitching was associated with increased kinetics of the upper extremity and decreased ball velocity (Fortenbaugh et al., 2009); future studies are required to investigate the movement patterns of pelvis, trunk, and upper extremities to understand the panorama among stride types.

### The Mechanism of the Kinematic Differences Among Three Stride Types in Stride Leg

We hypothesized that the DD pitchers would generate less brace movements by stride leg during the arm-cocking and acceleration phase, but we further found that there were no significant differences in kinematic parameters (including joint angle, angular velocity, timing of the peak value of joint angle, and angular velocity) of the hip, knee, and ankle joints of the stride leg among the three groups. Previous studies on the kinematic parameters of the stride leg that investigated the relationship between these parameters and ball velocity (Elliott et al., 1988; Matsuo et al., 2001; Escamilla et al., 2002; Kageyama et al., 2014) found that the stride leg contributes to trunk rotation in the arm-cocking phase and braces the trunk and upper extremities in the arm-acceleration phase. Also, extension of the stride knee in the arm-acceleration phase enhances the ball velocity. In contrast, we found no significant differences in ball velocity among the stride types, indicating that the movement patterns of the stride leg are not affected by the movement patterns of the pivot leg. The other research also proposed that maintaining a stable pivot leg by knee extension helps to transfer energy through the trunk to the throwing arm, which might be critical for maximizing the pitch velocity (Matsuo et al., 2001). Although we noted no significant differences between the three stride types in terms of ball velocity or kinematic variables of the stride leg, the stride legs of TF pitchers must brace the trunk and upper extremities together with pivot–hip adduction and pivot–knee extension during the arm-cocking/acceleration phases. In addition, the participants may not perform their usual movement of stride legs due to the laboratory setting, and the participants could not wear spikes as usual.

### The Definition of Stride Types

Based on the trajectory of the pelvis center, the participants were separated into three different groups. As the original definition, the TF pitchers positioned their body to be as tall as possible and take a “controlled fall” driving movement until their stride leg contacts the ground (Ryan and House, 1991). On the other hand, DD pitchers pushed off from the pitching rubber to drive the body forward, meaning that their movements are more parallel with the ground (Figures 2A,B). In the current study, we not just successfully identify TF and DD groups, but also categorize an additional MX group that includes pitchers whose pelvis center trajectory moved forward first, followed by a quick downward movement (Figure 2C), and the body sizes among these three group are the same. The previous study demonstrated that pitchers who come from different countries may be taught in different pitching techniques with anthropometry differences (Oi et al., 2019); however, our study supports that the difference existed among these three stride types that were caused by pitching technique instead of anthropometry in Taiwan.

### Limitations

All participants of the groups cannot wear spikes as they normally would wear on a baseball field. The force plates were also horizontally level with the floor, which somewhat differs from a pitching mound. The experimental space of current research was an indoor laboratory, so the pitching distance for the experiment is 7.5 m instead of the regular distance (18.44 m). Future studies could compare kinematic and kinetic differences in the trunk and upper limbs among stride types.

### Summary

Our study demonstrates the differences in stride coordination from the kinematics of the lower extremities between the stride types. For kinematics at the instantaneous time of the selected event, only angular velocity at the stride–foot contact of pivot leg was found to have greater hip adduction angular velocity on the frontal plane at DD and MX groups than at TF group as well as ankle plantar–flexion angular velocity. Regarding movement patterns, during the stride phase, DD group pitchers accelerated their body forward by more and earlier hip adduction, hip extension, hip external rotation, and knee extension, while MX group pitchers used more ankle plantar–flexion to acceleration their body forward. During the arm-cocking and acceleration phase, the movement patterns of the stride leg were similar among the three groups even though the movement patterns of the pivot leg were different in the stride phase. Interestingly, in contrast to the other two groups, the MX group, in starting forward then downward, had partial specific differences in stride patterns. This suggests that a MX type also exists, except for TF and DD stride types, exists in Taiwan, and may also exist in other countries.

## Conclusions

Based on the trajectory of the pelvis center during the stride phase, the stride types of baseball pitchers can be categorized into three types: TF, DD, and MX. The DD pitching movement involves early pivot–knee extension and pivot–hip adduction to move the body forward, whereas TF pitchers maintain forward acceleration using the pivot leg until the middle of the arm-cocking/acceleration phase. The MX pitchers were similar to DD pitchers in terms of movement patterns of the pivot leg during the stride phase. The findings presented here can be used to develop training strategies for coaches, players, and trainers to better meet the demands of different pitching styles.

## Data Availability Statement

The raw data supporting the conclusions of this article will be made available by the authors, without undue reservation.

## Ethics Statement

The studies involving human participants were reviewed and approved by Institutional Review Board of National Taiwan Sports University. The patients/participants provided their written informed consent to participate in this study.

## Author Contributions

S-WC ideated and operated at the experiment and wrote the manuscript. W-TT ideated and instructed the experiment and data processing & analysis and wrote the manuscript. J-TK ideated the experiment and coordinated the participants. T-YH, Y-LC, and W-HL collaborated in the experiment, data analysis and literature searching. DB collaborated in the experiment execution and English edition and revising. All authors have read and approved the final version of the manuscript.

## Conflict of Interest

The authors declare that the research was conducted in the absence of any commercial or financial relationships that could be construed as a potential conflict of interest.

## Publisher's Note

All claims expressed in this article are solely those of the authors and do not necessarily represent those of their affiliated organizations, or those of the publisher, the editors and the reviewers. Any product that may be evaluated in this article, or claim that may be made by its manufacturer, is not guaranteed or endorsed by the publisher.
