# Supplementary material for: Stride Pattern of the Lower Extremities Among Stride Types in Baseball Pitching
Source: Front Sports Act Living. 2021 Aug 2;3:670395. doi: 10.3389/fspor.2021.670395 (PMC8366559; doi:10.3389/fspor.2021.670395)
Supplement: Supplementary file 1 [file Data_Sheet_1.DOCX]

Appendix

Table App. 1 The kinematics variables of pivot-hip internal rotation and external rotation

| Variables | TF Group  (N=6) | | | DD Group  (N=12) | | | Mix Group  (N=12) | | | p Value | Comparison |
| --- | --- | --- | --- | --- | --- | --- | --- | --- | --- | --- | --- |
| *Hip Internal Rotation (+)/External Rotation (-) Angle of Pivot Leg* | | | | | | | | | | | |
| At SFC (^∘^) | 11.75 | ± | 10.28 | 14.56 | ± | 8.85 | 14.14 | ± | 12.30 | 0.864 |  |
| At MER (^∘^) | 12.89 | ± | 6.67 | 15.81 | ± | 8.74 | 21.33 | ± | 6.81 | 0.072 |  |
| At REL (^∘^) | 14.12 | ± | 8.83 | 15.82 | ± | 9.48 | 20.56 | ± | 7.25 | 0.245 |  |
| Max in PI (^∘^) | 21.52 | ± | 6.29 | 28.85 | ± | 10.81 | 24.03 | ± | 8.29 | 0.233 |  |
| MaxT in PI (%) | 52.40 | ± | 39.76 | 63.93 | ± | 35.80 | 62.88 | ± | 32.88 | 0.793 |  |
| Min in PI (^∘^) | 2.13 | ± | 1.21 | 3.30 | ± | 2.62 | 2.76 | ± | 2.03 | 0.558 |  |
| MinT in PI (%) | 58.53 | ± | 26.10 | 61.13 | ± | 22.94 | 53.00 | ± | 30.41 | 0.755 |  |
| Max in PII (^∘^) | 21.77 | ± | 3.78 | 23.00 | ± | 7.85 | 26.36 | ± | 7.55 | 0.360 |  |
| MaxT in PII (%) | 150.38 | ± | 45.44 | 158.80 | ± | 41.81 | 159.95 | ± | 38.49 | 0.890 |  |
| Min in PII (^∘^) | 1.87 | ± | 0.74 | 3.18 | ± | 2.59 | 4.01 | ± | 4.99 | 0.502 |  |
| MinT in PII (%) | 154.31 | ± | 26.05 | 133.38 | ± | 21.96 | 133.06 | ± | 20.75 | 0.136 |  |
| *Hip Internal Rotation (+)/External Rotation (-) Angular Velocity of Pivot Leg* | | | | | | | | | | | |
| At SFC (^∘^/s) | 42.99 | ± | 163.59 | -138.52 | ± | 261.02 | 32.72 | ± | 234.89 | 0.156 |  |
| At MER (^∘^/s) | 28.16 | ± | 133.41 | 36.82 | ± | 90.47 | 27.44 | ± | 129.84 | 0.978 |  |
| At REL (^∘^/s) | 75.50 | ± | 167.75 | -11.28 | ± | 123.39 | -51.03 | ± | 113.12 | 0.165 |  |
| Max in PI (^∘^/s) | 194.56 | ± | 140.31 | 231.00 | ± | 174.74 | 236.17 | ± | 190.29 | 0.886 |  |
| MaxT in PI (%) | 85.57 | ± | 14.71 | 82.29 | ± | 17.44 | 70.88 | ± | 30.90 | 0.423/0.304 |  |
| Min in PI (^∘^/s) | -160.21 | ± | 45.09 | -307.21 | ± | 139.87 | -233.38 | ± | 144.66 | 0.011* | DD>TF |
| MinT in PI (%) | 76.40 | ± | 29.59 | 88.28 | ± | 18.36 | 79.17 | ± | 25.65 | 0.522 |  |
| Max in PII (^∘^/s) | 339.07 | ± | 130.59 | 291.23 | ± | 126.40 | 368.37 | ± | 101.27 | 0.288 |  |
| MaxT in PII (%) | 140.62 | ± | 21.80 | 149.49 | ± | 21.00 | 147.41 | ± | 15.65 | 0.651 |  |
| Min in PII (^∘^/s) | -316.59 | ± | 110.94 | -348.82 | ± | 155.82 | -316.36 | ± | 203.57 | 0.879 |  |
| MinT in PII (%) | 137.51 | ± | 26.28 | 124.56 | ± | 28.01 | 151.82 | ± | 31.95 | 0.094 |  |

SFC: Stride foot contact the ground; MER: Maximum of shoulder external rotation; REL: Ball released.; Max: Maximum; Min: Minimum; PI: Stride Phase; PII: Arm Cocking and Acceleration Phase.

Table App. 2 The kinematics variables of pivot-hip abduction and adduction

| Variables | TF Group  (N=6) | | | DD Group  (N=12) | | | Mix Group  (N=12) | | | p Value | Comparison |
| --- | --- | --- | --- | --- | --- | --- | --- | --- | --- | --- | --- |
| *Hip Abduction (+)/Adduction (-) Angle (^∘^) of Pivot Leg* | | | | | | | | | | | |
| At SFC (^∘^) | 25.22 | ± | 10.84 | 22.88 | ± | 7.30 | 24.07 | ± | 5.97 | 0.820 |  |
| At MER (^∘^) | 0.62 | ± | 5.40 | 0.30 | ± | 7.09 | -0.65 | ± | 4.06 | 0.878 |  |
| At REL (^∘^) | 1.56 | ± | 4.93 | 1.63 | ± | 6.93 | -0.60 | ± | 4.55 | 0.589 |  |
| Max in PI (^∘^) | 27.35 | ± | 10.56 | 30.82 | ± | 10.12 | 30.86 | ± | 5.67 | 0.683 |  |
| MaxT in PI (%) | 95.03 | ± | 4.34 | 84.63 | ± | 11.29 | 90.10 | ± | 5.34 | 0.047 |  |
| Min in PI (^∘^) | 5.88 | ± | 9.45 | 11.67 | ± | 6.63 | 8.77 | ± | 5.11 | 0.228 |  |
| MinT in PI (%) | 27.63 | ± | 15.80 | 26.16 | ± | 31.25 | 30.66 | ± | 30.59 | 0.928 |  |
| Max in PII (^∘^) | 25.24 | ± | 10.87 | 22.98 | ± | 7.13 | 24.08 | ± | 5.98 | 0.832 |  |
| MaxT in PII (%) | 101.11 | ± | 2.72 | 101.42 | ± | 4.93 | 100.45 | ± | 1.56 | 0.790 |  |
| Min in PII (^∘^) | -0.18 | ± | 4.92 | -0.42 | ± | 7.14 | -1.28 | ± | 4.27 | 0.905 |  |
| MinT in PII (%) | 191.22 | ± | 9.21 | 180.62 | ± | 12.30 | 191.27 | ± | 10.52 | 0.051 |  |
| *Hip Abduction (+)/Adduction (-) Angular Velocity (^∘^/s) of Pivot Leg* | | | | | | | | | | | |
| At SFC (^∘^/s) | -66.20 | ± | 41.81 | -166.95 | ± | 107.07 | -154.83 | ± | 116.46 | 0.014* | DD>TF |
| At MER (^∘^/s) | -10.14 | ± | 76.14 | -3.82 | ± | 92.84 | -17.43 | ± | 48.17 | 0.905 |  |
| At REL (^∘^/s) | 68.88 | ± | 127.35 | 64.02 | ± | 74.95 | 10.67 | ± | 55.78 | 0.208 |  |
| Max in PI (^∘^/s) | 96.18 | ± | 51.93 | 93.25 | ± | 19.05 | 112.65 | ± | 35.01 | 0.301 |  |
| MaxT in PI (%) | 72.09 | ± | 15.14 | 63.29 | ± | 10.88 | 70.70 | ± | 15.15 | 0.308 |  |
| Min in PI (^∘^/s) | -78.84 | ± | 34.68 | -177.15 | ± | 88.15 | -168.68 | ± | 105.02 | 0.004* | DD>TF, Mix>TF |
| MinT in PI (%) | 73.47 | ± | 40.20 | 93.80 | ± | 20.38 | 90.53 | ± | 19.54 | 0.540 |  |
| Max in PII (^∘^/s) | 81.55 | ± | 114.70 | 72.82 | ± | 73.13 | 20.49 | ± | 54.59 | 0.167 |  |
| MaxT in PII (%) | 181.19 | ± | 39.78 | 185.44 | ± | 27.37 | 185.11 | ± | 27.82 | 0.957 |  |
| Min in PII (^∘^/s) | -252.67 | ± | 61.63 | -285.69 | ± | 87.56 | -289.18 | ± | 63.56 | 0.591 |  |
| MinT in PII (%) | 155.46 | ± | 19.77 | 132.12 | ± | 15.98 | 131.45 | ± | 19.67 | 0.029* | TF>Mix |

SFC: Stride foot contact the ground; MER: Maximum of shoulder external rotation; REL: Ball released.; Max: Maximum; Min: Minimum; PI: Stride Phase; PII: Arm Cocking and Acceleration Phase.

Table App. 3 The kinematics variables of pivot-hip extansion and flexion

| Variables | TF Group  (N=6) | | | DD Group  (N=12) | | | Mix Group  (N=12) | | | p Value | Comparison |
| --- | --- | --- | --- | --- | --- | --- | --- | --- | --- | --- | --- |
| *Hip Extension (+)/Flexion (-) Angle (^∘^) of Pivot Leg* | | | | | | | | | | | |
| At SFC (^∘^) | 81.33 | ± | 14.15 | 94.83 | ± | 12.64 | 90.13 | ± | 14.03 | 0.155 |  |
| At MER (^∘^) | 105.34 | ± | 4.42 | 103.56 | ± | 5.53 | 102.61 | ± | 6.82 | 0.659 |  |
| At REL (^∘^) | 100.18 | ± | 5.85 | 95.68 | ± | 7.26 | 96.53 | ± | 8.02 | 0.469 |  |
| Max in PI (^∘^) | 105.79 | ± | 5.81 | 99.79 | ± | 8.81 | 103.95 | ± | 7.24 | 0.241 |  |
| MaxT in PI (%) | 0.00 | ± | 0.00 | 58.33 | ± | 51.49 | 16.67 | ± | 38.92 | 0.140 |  |
| Min in PI (^∘^) | 57.65 | ± | 11.05 | 56.56 | ± | 11.97 | 51.82 | ± | 10.15 | 0.468 |  |
| MinT in PI (%) | 79.05 | ± | 3.69 | 68.20 | ± | 10.12 | 73.30 | ± | 4.92 | 0.021* | TF>DD |
| Max in PII (^∘^) | 112.81 | ± | 4.15 | 110.45 | ± | 5.27 | 110.67 | ± | 5.74 | 0.649 |  |
| MaxT in PII (%) | 155.55 | ± | 9.97 | 144.76 | ± | 12.01 | 145.40 | ± | 13.46 | 0.193 |  |
| Min in PII (^∘^) | 81.32 | ± | 14.15 | 90.43 | ± | 11.17 | 86.15 | ± | 10.77 | 0.297 |  |
| MinT in PII (%) | 116.67 | ± | 40.82 | 150.00 | ± | 52.22 | 150.00 | ± | 52.22 | 0.286 |  |
| *Hip Extension (+)/Flexion (-) Angular Velocity (^∘^/s) of Pivot Leg* | | | | | | | | | | | |
| At SFC (^∘^/s) | 374.54 | ± | 91.34 | 352.00 | ± | 100.54 | 372.90 | ± | 158.72 | 0.901 |  |
| At MER (^∘^/s) | -182.37 | ± | 84.74 | -204.50 | ± | 70.21 | -206.42 | ± | 89.93 | 0.824 |  |
| At REL (^∘^/s) | -183.99 | ± | 70.43 | -233.49 | ± | 91.25 | -231.38 | ± | 89.72 | 0.485 |  |
| Max in PI (^∘^/s) | 376.87 | ± | 92.48 | 408.27 | ± | 107.12 | 437.62 | ± | 159.43 | 0.635 |  |
| MaxT in PI (%) | 99.64 | ± | 0.61 | 96.79 | ± | 3.62 | 97.88 | ± | 2.28 | 0.011* | TF>DD |
| Min in PI (^∘^/s) | -136.97 | ± | 32.18 | -142.65 | ± | 53.68 | -158.27 | ± | 33.92 | 0.537 |  |
| MinT in PI (%) | 35.39 | ± | 17.69 | 13.68 | ± | 9.85 | 24.82 | ± | 14.71 | 0.011* |  |
| Max in PII (^∘^/s) | 449.76 | ± | 105.84 | 359.14 | ± | 98.20 | 408.44 | ± | 171.03 | 0.385 |  |
| MaxT in PII (%) | 108.75 | ± | 10.81 | 102.09 | ± | 4.57 | 104.68 | ± | 9.09 | 0.265 |  |
| Min in PII (^∘^/s) | -226.66 | ± | 81.36 | -251.18 | ± | 77.45 | -246.57 | ± | 78.36 | 0.818 |  |
| MinT in PII (%) | 187.12 | ± | 9.12 | 190.64 | ± | 6.76 | 191.69 | ± | 7.33 | 0.477 |  |

SFC: Stride foot contact the ground; MER: Maximum of shoulder external rotation; REL: Ball released.; Max: Maximum; Min: Minimum; PI: Stride Phase; PII: Arm Cocking and Acceleration Phase.

Table App. 4 The kinematics variables of pivot-knee extansion and flexion

| Variables | TF Group  (N=6) | | | DD Group  (N=12) | | | Mix Group  (N=12) | | | p Value | Comparison |
| --- | --- | --- | --- | --- | --- | --- | --- | --- | --- | --- | --- |
| *Knee Extension (+)/Flexion (-) Angle (^∘^) of Pivot Leg* | | | | | | | | | | | |
| At SFC (^∘^) | 125.93 | ± | 7.24 | 133.97 | ± | 12.44 | 130.48 | ± | 8.59 | 0.290 |  |
| At MER (^∘^) | 138.17 | ± | 5.97 | 140.29 | ± | 11.37 | 130.69 | ± | 9.35 | 0.054 |  |
| At REL (^∘^) | 132.05 | ± | 7.60 | 131.71 | ± | 15.19 | 122.45 | ± | 5.83 | 0.096 |  |
| Max in PI (^∘^) | 157.82 | ± | 4.53 | 152.09 | ± | 5.08 | 151.14 | ± | 5.83 | 0.047 |  |
| MaxT in PI (%) | 0.00 | ± | 0.00 | 0.00 | ± | 0.00 | 9.44 | ± | 27.93 | ---- |  |
| Min in PI (^∘^) | 120.60 | ± | 6.01 | 114.33 | ± | 9.10 | 112.41 | ± | 7.88 | 0.139 |  |
| MinT in PI (%) | 89.65 | ± | 7.58 | 69.43 | ± | 7.92 | 77.28 | ± | 3.84 | 0.000* | TF>DD, TF>Mix, Mix>DD |
| Max in PII (^∘^) | 146.64 | ± | 6.30 | 149.34 | ± | 6.98 | 144.51 | ± | 5.51 | 0.175 |  |
| MaxT in PII (%) | 156.56 | ± | 12.34 | 150.85 | ± | 17.73 | 146.02 | ± | 17.29 | 0.439 |  |
| Min in PII (^∘^) | 123.66 | ± | 7.21 | 126.32 | ± | 12.15 | 119.68 | ± | 7.80 | 0.272 |  |
| MinT in PII (%) | 133.33 | ± | 51.64 | 141.67 | ± | 51.49 | 169.23 | ± | 48.04 | 0.254 |  |
| *Knee Extension (+)/Flexion (-) Angular Velocity (^∘^/s) of Pivot Leg* | | | | | | | | | | | |
| At SFC (^∘^/s) | 121.31 | ± | 39.86 | 191.50 | ± | 120.23 | 200.11 | ± | 82.19 | 0.230 |  |
| At MER (^∘^/s) | -230.73 | ± | 128.68 | -252.80 | ± | 155.86 | -336.85 | ± | 104.97 | 0.170 |  |
| At REL (^∘^/s) | -234.73 | ± | 131.48 | -241.25 | ± | 140.00 | -279.36 | ± | 59.16 | 0.676 |  |
| Max in PI (^∘^/s) | 121.97 | ± | 40.98 | 211.16 | ± | 103.59 | 221.00 | ± | 59.16 | 0.002* | DD>TF, Mix>TF |
| MaxT in PI (%) | 99.71 | ± | 0.71 | 96.33 | ± | 6.98 | 98.79 | ± | 2.46 | 0.172 |  |
| Min in PI (^∘^/s) | -93.60 | ± | 20.74 | -120.02 | ± | 28.87 | -117.65 | ± | 37.30 | 0.230 |  |
| MinT in PI (%) | 42.38 | ± | 26.53 | 23.24 | ± | 16.80 | 42.23 | ± | 14.88 | 0.029* | Mix>DD |
| Max in PII (^∘^/s) | 261.55 | ± | 79.67 | 259.29 | ± | 119.16 | 238.72 | ± | 87.73 | 0.843 |  |
| MaxT in PII (%) | 30.11 | ± | 11.48 | 18.14 | ± | 15.34 | 14.13 | ± | 14.51 | 0.095 |  |
| Min in PII (^∘^/s) | -267.15 | ± | 108.98 | -283.17 | ± | 142.26 | -359.98 | ± | 93.14 | 0.171 |  |
| MinT in PII (%) | 86.01 | ± | 11.25 | 83.37 | ± | 9.56 | 82.00 | ± | 7.75 | 0.680 |  |

SFC: Stride foot contact the ground; MER: Maximum of shoulder external rotation; REL: Ball released.; Max: Maximum; Min: Minimum; PI: Stride Phase; PII: Arm Cocking and Acceleration Phase.

Table App. 5 The kinematics variables of pivot-ankle plantar flexion and dorsi flexion

| Variables | TF Group  (N=6) | | | DD Group  (N=12) | | | Mix Group  (N=12) | | | p Value | Comparison |
| --- | --- | --- | --- | --- | --- | --- | --- | --- | --- | --- | --- |
| *Ankle Plantar Flexion Angle (^∘^) of Pivot Leg* | | | | | | | | | | | |
| At SFC (^∘^) | 91.00 | ± | 16.99 | 109.25 | ± | 20.44 | 103.28 | ± | 13.91 | 0.126 |  |
| At MER (^∘^) | 121.11 | ± | 15.08 | 132.25 | ± | 12.35 | 124.65 | ± | 8.69 | 0.119 |  |
| At REL (^∘^) | 121.12 | ± | 14.70 | 132.24 | ± | 12.54 | 125.55 | ± | 13.64 | 0.114 |  |
| Max in PI (^∘^) | 95.78 | ± | 10.93 | 109.63 | ± | 20.07 | 103.54 | ± | 13.64 | 0.238 |  |
| MaxT in PI (%) | 66.67 | ± | 51.64 | 91.67 | ± | 28.87 | 94.34 | ± | 20.40 | 0.194 |  |
| Min in PI (^∘^) | 75.40 | ± | 4.98 | 74.06 | ± | 9.11 | 76.78 | ± | 7.55 | 0.690 |  |
| MinT in PI (%) | 64.16 | ± | 35.35 | 54.29 | ± | 16.26 | 60.97 | ± | 28.22 | 0.703 |  |
| Max in PII (^∘^) | 126.35 | ± | 16.54 | 136.61 | ± | 11.80 | 131.68 | ± | 9.68 | 0.235 |  |
| MaxT in PII (%) | 166.59 | ± | 17.75 | 159.72 | ± | 19.59 | 158.27 | ± | 20.83 | 0.691 |  |
| Min in PII (^∘^) | 91.00 | ± | 16.99 | 109.25 | ± | 20.44 | 103.28 | ± | 13.91 | 0.126 |  |
| MinT in PII (%) | 100.00 | ± | 0.00 | 100.00 | ± | 0.00 | 100.00 | ± | 0.00 | ---- |  |
| *Ankle Plantar Flexion (+) Dorsi Flexion (-) Angular Velocity (^∘^/s) of Pivot Leg* | | | | | | | | | | | |
| At SFC (^∘^/s) | 225.39 | ± | 152.31 | 345.17 | ± | 107.45 | 388.93 | ± | 127.04 | 0.043* | Mix>TF |
| At MER (^∘^/s) | -63.88 | ± | 94.49 | -53.98 | ± | 93.24 | -58.63 | ± | 87.29 | 0.976 |  |
| At REL (^∘^/s) | 21.61 | ± | 95.33 | 16.44 | ± | 123.36 | 81.67 | ± | 124.90 | 0.292 |  |
| Max in PI (^∘^/s) | 225.39 | ± | 152.31 | 368.46 | ± | 112.07 | 393.73 | ± | 124.90 | 0.033* | Mix>TF |
| MaxT in PI (%) | 100.00 | ± | 0.00 | 98.75 | ± | 1.81 | 99.57 | ± | 1.56 | ---- |  |
| Min in PI (^∘^/s) | -47.24 | ± | 20.69 | -51.65 | ± | 20.23 | -49.66 | ± | 19.14 | 0.904 |  |
| MinT in PI (%) | 54.87 | ± | 16.58 | 24.17 | ± | 20.39 | 38.27 | ± | 21.37 | 0.017* | TF>DD |
| Max in PII (^∘^/s) | 437.95 | ± | 203.63 | 400.07 | ± | 116.70 | 452.66 | ± | 141.64 | 0.663 |  |
| MaxT in PII (%) | 131.23 | ± | 18.11 | 114.71 | ± | 18.89 | 112.01 | ± | 13.43 | 0.072 |  |
| Min in PII (^∘^/s) | -195.44 | ± | 145.90 | -175.55 | ± | 88.70 | -221.37 | ± | 192.98 | 0.752 |  |
| MinT in PII (%) | 176.50 | ± | 13.25 | 177.27 | ± | 12.84 | 169.53 | ± | 9.78 | 0.230 |  |

SFC: Stride foot contact the ground; MER: Maximum of shoulder external rotation; REL: Ball released.; Max: Maximum; Min: Minimum; PI: Stride Phase; PII: Arm Cocking and Acceleration Phase.

Table App. 6 The kinematics variables of stride-hip internal rotation and external rotation

| Variables | TF Group  (N=6) | | | DD Group  (N=12) | | | Mix Group  (N=12) | | | p Value | Comparison |
| --- | --- | --- | --- | --- | --- | --- | --- | --- | --- | --- | --- |
| *Hip Internal Rotation (+)/External Rotation (-) Angle (^∘^) of Stride Leg* | | | | | | | | | | | |
| At SFC (^∘^) | 57.17 | ± | 9.44 | 45.04 | ± | 10.96 | 51.09 | ± | 11.84 | 0.099 |  |
| At MER (^∘^) | 6.21 | ± | 4.09 | 9.99 | ± | 8.60 | 8.66 | ± | 8.40 | 0.635 |  |
| At REL (^∘^) | 5.85 | ± | 3.59 | 11.95 | ± | 7.46 | 9.00 | ± | 7.90 | 0.234 |  |
| Max in PII (^∘^) | 58.27 | ± | 7.90 | 45.52 | ± | 11.12 | 51.72 | ± | 11.49 | 0.070 |  |
| MaxT in PII (%) | 102.44 | ± | 5.00 | 102.06 | ± | 4.33 | 103.29 | ± | 5.08 | 0.818 |  |
| Min in PII (^∘^) | 1.47 | ± | 0.24 | 1.88 | ± | 1.01 | 3.08 | ± | 4.21 | 0.426 |  |
| MinT in PII (%) | 179.95 | ± | 17.32 | 169.67 | ± | 13.34 | 181.75 | ± | 15.58 | 0.140 |  |
| *Hip Internal Rotation (+)/External Rotation (-) Angular Velocity (^∘^/s) of Stride Leg* | | | | | | | | | | | |
| At SFC (^∘^/s) | -22.26 | ± | 233.75 | -142.00 | ± | 242.17 | -35.74 | ± | 205.93 | 0.431 |  |
| At MER (^∘^/s) | -24.88 | ± | 154.54 | 108.51 | ± | 218.70 | -76.28 | ± | 184.31 | 0.077 |  |
| At REL (^∘^/s) | -16.85 | ± | 96.02 | 48.84 | ± | 156.04 | 17.35 | ± | 113.22 | 0.594 |  |
| Max in PII (^∘^/s) | 202.53 | ± | 200.14 | 333.36 | ± | 202.79 | 228.25 | ± | 230.18 | 0.365 |  |
| MaxT in PII (%) | 152.08 | ± | 41.86 | 168.50 | ± | 23.89 | 161.81 | ± | 39.28 | 0.637 |  |
| Min in PII (^∘^/s) | -583.36 | ± | 82.79 | -526.27 | ± | 77.27 | -558.94 | ± | 162.28 | 0.395 |  |
| MinT in PII (%) | 144.02 | ± | 15.17 | 139.91 | ± | 8.59 | 146.45 | ± | 9.99 | 0.332 |  |

SFC: Stride foot contact the ground; MER: Maximum of shoulder external rotation; REL: Ball released.; Max: Maximum; Min: Minimum; PI: Stride Phase; PII: Arm Cocking and Acceleration Phase.

Table App. 7 The kinematics variables of stride-hip abduction and adduction

| Variables | TF Group  (N=6) | | | DD Group  (N=12) | | | Mix Group  (N=12) | | | p Value | Comparison |
| --- | --- | --- | --- | --- | --- | --- | --- | --- | --- | --- | --- |
| *Hip Abduction (+)/Adduction (-) Angle (^∘^) of Stride Leg* | | | | | | | | | | | |
| At SFC (^∘^) | -39.96 | ± | 3.07 | -41.59 | ± | 9.45 | -43.45 | ± | 5.60 | 0.271 |  |
| At MER (^∘^) | 55.35 | ± | 23.54 | 46.27 | ± | 20.88 | 44.96 | ± | 21.35 | 0.612 |  |
| At REL (^∘^) | 57.82 | ± | 18.10 | 56.10 | ± | 17.24 | 49.02 | ± | 16.62 | 0.488 |  |
| Max in PII (^∘^) | 60.50 | ± | 21.42 | 56.92 | ± | 18.26 | 51.84 | ± | 19.13 | 0.641 |  |
| MaxT in PII (%) | 195.08 | ± | 7.76 | 195.87 | ± | 6.86 | 190.79 | ± | 10.91 | 0.356 |  |
| Min in PII (^∘^) | -44.50 | ± | 5.73 | -44.83 | ± | 12.18 | -48.34 | ± | 10.10 | 0.650 |  |
| MinT in PII (%) | 116.72 | ± | 16.28 | 116.45 | ± | 18.88 | 120.38 | ± | 19.07 | 0.857 |  |
| *Hip Abduction (+)/Adduction (-) Angular Velocity (^∘^/s) of Stride Leg* | | | | | | | | | | | |
| At SFC (^∘^/s) | -101.20 | ± | 140.16 | -28.49 | ± | 117.98 | -87.97 | ± | 101.04 | 0.342 |  |
| At MER (^∘^/s) | 218.43 | ± | 388.64 | 698.80 | ± | 685.51 | 387.18 | ± | 454.21 | 0.186 |  |
| At REL (^∘^/s) | -46.96 | ± | 265.08 | 78.76 | ± | 151.60 | 34.99 | ± | 276.37 | 0.561 |  |
| Max in PII (^∘^/s) | 2264.67 | ± | 1290.86 | 2180.51 | ± | 1167.74 | 2321.48 | ± | 1261.19 | 0.773 |  |
| MaxT in PII (%) | 160.00 | ± | 8.79 | 160.61 | ± | 7.70 | 162.77 | ± | 5.64 | 0.670 |  |
| Min in PII (^∘^/s) | -143.62 | ± | 188.83 | -70.28 | ± | 150.12 | -343.79 | ± | 675.23 | 0.328 |  |
| MinT in PII (%) | 148.46 | ± | 53.09 | 131.85 | ± | 46.09 | 138.56 | ± | 47.42 | 0.787 |  |

SFC: Stride foot contact the ground; MER: Maximum of shoulder external rotation; REL: Ball released.; Max: Maximum; Min: Minimum; PI: Stride Phase; PII: Arm Cocking and Acceleration Phase.

Table App. 8 The kinematics variables of stride-hip extansion and flexion

| Variables | TF Group  (N=6) | | | DD Group  (N=12) | | | Mix Group  (N=12) | | | p Value | Comparison |
| --- | --- | --- | --- | --- | --- | --- | --- | --- | --- | --- | --- |
| *Hip Extension (+)/Flexion (-)Angle (^∘^) of Stride Leg* | | | | | | | | | | | |
| At SFC (^∘^) | 65.63 | ± | 10.09 | 56.25 | ± | 14.70 | 56.88 | ± | 11.13 | 0.299 |  |
| At MER (^∘^) | 13.63 | ± | 8.89 | 16.13 | ± | 11.11 | 15.55 | ± | 7.36 | 0.865 |  |
| At REL (^∘^) | 13.85 | ± | 7.65 | 16.55 | ± | 11.54 | 17.76 | ± | 8.58 | 0.728 |  |
| Max in PII (^∘^) | 65.63 | ± | 10.09 | 56.25 | ± | 14.70 | 56.88 | ± | 11.13 | 0.299 |  |
| MaxT in PII (%) | 100.00 | ± | 0.00 | 100.00 | ± | 0.00 | 100.00 | ± | 0.00 | --- |  |
| Min in PII (^∘^) | 11.97 | ± | 7.94 | 14.63 | ± | 10.78 | 14.12 | ± | 7.69 | 0.839 |  |
| MinT in PII (%) | 186.71 | ± | 11.65 | 178.56 | ± | 14.58 | 178.31 | ± | 14.00 | 0.435 |  |
| *Hip Extension (+)/Flexion (-) Angular Velocity (^∘^/s) of Stride Leg* | | | | | | | | | | | |
| At SFC (^∘^/s) | -379.02 | ± | 96.25 | -380.34 | ± | 120.57 | -358.84 | ± | 152.77 | 0.911 |  |
| At MER (^∘^/s) | -4.61 | ± | 103.42 | 8.09 | ± | 94.10 | 55.34 | ± | 73.30 | 0.297 |  |
| At REL (^∘^/s) | 45.76 | ± | 115.70 | 27.37 | ± | 97.15 | 80.94 | ± | 113.59 | 0.478 |  |
| Max in PII (^∘^/s) | 70.89 | ± | 89.09 | 64.36 | ± | 60.92 | 122.64 | ± | 99.35 | 0.215 |  |
| MaxT in PII (%) | 186.63 | ± | 15.20 | 189.35 | ± | 8.20 | 191.05 | ± | 9.69 | 0.795 |  |
| Min in PII (^∘^/s) | -557.27 | ± | 119.37 | -532.94 | ± | 98.74 | -536.06 | ± | 114.15 | 0.899 |  |
| MinT in PII (%) | 127.41 | ± | 8.45 | 122.18 | ± | 12.48 | 125.95 | ± | 11.56 | 0.594 |  |

SFC: Stride foot contact the ground; MER: Maximum of shoulder external rotation; REL: Ball released.; Max: Maximum; Min: Minimum; PI: Stride Phase; PII: Arm Cocking and Acceleration Phase.

Table App. 9 The kinematics variables of stride -knee extansion and flexion

| Variables | TF Group  (N=6) | | | DD Group  (N=12) | | | Mix Group  (N=12) | | | p Value | Comparison |
| --- | --- | --- | --- | --- | --- | --- | --- | --- | --- | --- | --- |
| *Knee Extension Angle (^∘^) of Stride Leg* | | | | | | | | | | | |
| At SFC (^∘^) | 137.60 | ± | 8.88 | 127.18 | ± | 8.77 | 129.04 | ± | 9.24 | 0.077 |  |
| At MER (^∘^) | 125.10 | ± | 6.71 | 127.95 | ± | 14.81 | 126.90 | ± | 9.52 | 0.886 |  |
| At REL (^∘^) | 126.87 | ± | 8.62 | 133.45 | ± | 17.56 | 130.89 | ± | 9.05 | 0.637 |  |
| Max in PII (^∘^) | 140.32 | ± | 5.36 | 136.65 | ± | 13.98 | 134.78 | ± | 7.82 | 0.398 |  |
| MaxT in PII (%) | 116.67 | ± | 40.82 | 175.64 | ± | 44.11 | 161.54 | ± | 50.64 | 0.053 |  |
| Min in PII (^∘^) | 120.48 | ± | 3.12 | 121.18 | ± | 10.68 | 120.39 | ± | 8.53 | 0.204 |  |
| MinT in PII (%) | 158.30 | ± | 24.05 | 135.38 | ± | 32.80 | 139.43 | ± | 28.38 | 0.299 |  |
| *Knee Extension (+)/Flexion (-) Angular Velocity (^∘^/s) of Stride Leg* | | | | | | | | | | | |
| At SFC (^∘^/s) | -198.57 | ± | 83.70 | -45.34 | ± | 134.95 | -73.50 | ± | 177.83 | 0.126 |  |
| At MER (^∘^/s) | 68.04 | ± | 118.90 | 163.08 | ± | 135.08 | 126.48 | ± | 88.66 | 0.266 |  |
| At REL (^∘^/s) | 82.48 | ± | 107.22 | 178.09 | ± | 137.47 | 160.29 | ± | 84.15 | 0.252 |  |
| Max in PII (^∘^/s) | 123.82 | ± | 101.18 | 193.51 | ± | 130.76 | 191.72 | ± | 103.55 | 0.429 |  |
| MaxT in PII (%) | 141.20 | ± | 32.49 | 156.34 | ± | 35.73 | 135.79 | ± | 35.75 | 0.346 |  |
| Min in PII (^∘^/s) | -238.50 | ± | 94.07 | -149.12 | ± | 89.92 | -177.39 | ± | 105.41 | 0.205 |  |
| MinT in PII (%) | 124.82 | ± | 16.92 | 121.92 | ± | 26.28 | 129.42 | ± | 20.47 | 0.705 |  |

SFC: Stride foot contact the ground; MER: Maximum of shoulder external rotation; REL: Ball released.; Max: Maximum; Min: Minimum; PI: Stride Phase; PII: Arm Cocking and Acceleration Phase.

Table App. 10 The kinematics variables of stride -ankle plantar flexion and dorsi flexion

| Variables | TF Group  (N=6) | | | DD Group  (N=12) | | | Mix Group  (N=12) | | | p Value | Comparison |
| --- | --- | --- | --- | --- | --- | --- | --- | --- | --- | --- | --- |
| *Ankle Plantar Flexion Angle (^∘^) of Stride Leg* | | | | | | | | | | | |
| At SFC (^∘^) | 107.12 | ± | 10.04 | 106.80 | ± | 13.14 | 108.32 | ± | 13.80 | 0.955 |  |
| At MER (^∘^) | 101.09 | ± | 10.85 | 103.31 | ± | 14.61 | 105.14 | ± | 8.70 | 0.777 |  |
| At REL (^∘^) | 101.96 | ± | 11.15 | 105.10 | ± | 14.53 | 106.22 | ± | 13.48 | 0.766 |  |
| Max in PII (^∘^) | 111.85 | ± | 9.07 | 112.39 | ± | 11.04 | 114.98 | ± | 8.38 | 0.730 |  |
| MaxT in PII (%) | 140.18 | ± | 48.99 | 135.11 | ± | 46.77 | 141.00 | ± | 49.07 | 0.951 |  |
| Min in PII (^∘^) | 96.12 | ± | 7.38 | 96.95 | ± | 13.24 | 98.78 | ± | 8.04 | 0.844 |  |
| MinT in PII (%) | 138.28 | ± | 39.55 | 138.42 | ± | 36.14 | 133.89 | ± | 36.46 | 0.946 |  |
| *Ankle Plantar Flexion (+) Dorsi Flexion (-) Angular Velocity (^∘^/s) of Stride Leg* | | | | | | | | | | | |
| At SFC (^∘^/s) | -49.60 | ± | 190.31 | -52.70 | ± | 196.91 | 7.95 | ± | 222.69 | 0.734 |  |
| At MER (^∘^/s) | 25.23 | ± | 31.47 | 61.36 | ± | 56.90 | 26.17 | ± | 37.21 | 0.122 |  |
| At REL (^∘^/s) | 41.27 | ± | 40.11 | 52.08 | ± | 76.74 | 47.24 | ± | 67.73 | 0.932 |  |
| Max in PII (^∘^/s) | 123.95 | ± | 117.17 | 156.04 | ± | 114.54 | 177.13 | ± | 112.58 | 0.642 |  |
| MaxT in PII (%) | 141.20 | ± | 32.49 | 156.34 | ± | 35.73 | 135.79 | ± | 35.75 | 0.346 |  |
| Min in PII (^∘^/s) | -266.12 | ± | 258.36 | -233.59 | ± | 174.42 | -251.54 | ± | 232.84 | 0.952 |  |
| MinT in PII (%) | 124.82 | ± | 16.92 | 121.92 | ± | 26.28 | 129.42 | ± | 20.47 | 0.705 |  |

SFC: Stride foot contact the ground; MER: Maximum of shoulder external rotation; REL: Ball released.; Max: Maximum; Min: Minimum; PI: Stride Phase; PII: Arm Cocking and Acceleration Phase.
